# Detection and quantification of mitochondrial DNA deletions from next-generation sequence data

**DOI:** 10.1186/s12859-017-1821-7

**Published:** 2017-10-16

**Authors:** Colleen M. Bosworth, Sneha Grandhi, Meetha P. Gould, Thomas LaFramboise

**Affiliations:** Department of Genetics and Genome Sciences, Case Western Reserve University School of Medicine, Cleveland, OH 44106 USA

**Keywords:** Next-generation sequencing, Mitochondria DNA, Human genome, Chromosomal deletions

## Abstract

**Background:**

Chromosomal deletions represent an important class of human genetic variation. Various methods have been developed to mine “next-generation” sequencing (NGS) data to detect deletions and quantify their clonal abundances. These methods have focused almost exclusively on the nuclear genome, ignoring the mitochondrial chromosome (mtDNA). Detecting mtDNA deletions requires special care. First, the chromosome’s relatively small size (16,569 bp) necessitates the ability to detect extremely focal events. Second, the chromosome can be present at thousands of copies in a single cell (in contrast to two copies of nuclear chromosomes), and mtDNA deletions may be present on only a very small percentage of chromosomes. Here we present a method, termed MitoDel, to detect mtDNA deletions from NGS data.

**Results:**

We validate the method on simulated and real data, and show that MitoDel can detect novel and previously-reported mtDNA deletions. We establish that MitoDel can find deletions such as the “common deletion” at heteroplasmy levels well below 1%.

**Conclusions:**

MitoDel is a tool for detecting large mitochondrial deletions at low heteroplasmy levels. The tool can be downloaded at http://mendel.gene.cwru.edu/laframboiselab/.

## Background

Human genetic variation takes many forms, including single nucleotide variants, small insertions/deletions, larger chromosomal gains and losses, and inter-chromosomal translocations. A central pursuit in biomedical research is to determine those variants associated with human disease. Technological advances over the past several years have facilitated studies examining genetic variation at ever-increasing resolution, allowing better identification of variant-disease connections. Robust and accurate algorithms to detect all forms of human genetic variation from the ever-increasing number of large genomic data sets are necessary.

The vast majority of human DNA variant-detection algorithms focus exclusively on the 24 chromosomes (22 autosomes, X, and Y) comprising the nuclear genome. Usually ignored is the mitochondrial genome, despite the role of the mitochondrion in cellular bioenergetics and the known importance of mitochondrial mutations in a number of human diseases [1–7]. The mitochondrial genome (mtDNA) has features that distinguish it from its more commonly studied nuclear counterpart. First, the nuclear autosomal chromosomes are normally present in two copies per cell, while the number of copies of the mitochondrial chromosome varies widely from cell to cell, largely depending on tissue type. The mitochondrial chromosome may be present at hundreds, thousands, or tens of thousands of copies in a cell [8]. Second, the mutation rate of the mitochondrial genome is much higher than that of the nuclear genome and its repair mechanisms are far inferior to those in the nucleus [9]. The cell therefore carries considerably more mtDNA variants – both inherited and acquired – per base position than nuclear variants. Third, the mitochondrial genome is much smaller (16,569 bp) than the nuclear genome (~3.2 billion bp) and is circular rather than linear. Finally, mtDNA inheritance is strictly maternal. All of these differences present opportunities and challenges from an analytic perspective.

Since established computational tools used to identify biologically important nuclear DNA variants are often not adaptable to the mitochondrial genome, it is vitally important to develop new approaches to assess and quantify mtDNA genomic variation. Robust assessment of this variation in humans will allow identification of those variants that drive phenotypes, both benign and pathogenic. Owing to the limitations of established methods, this will necessitate the formulation of novel approaches particularly suited to the unique data types and biological scenarios inherent to mitochondrial genomics.

**Fig. 1:**
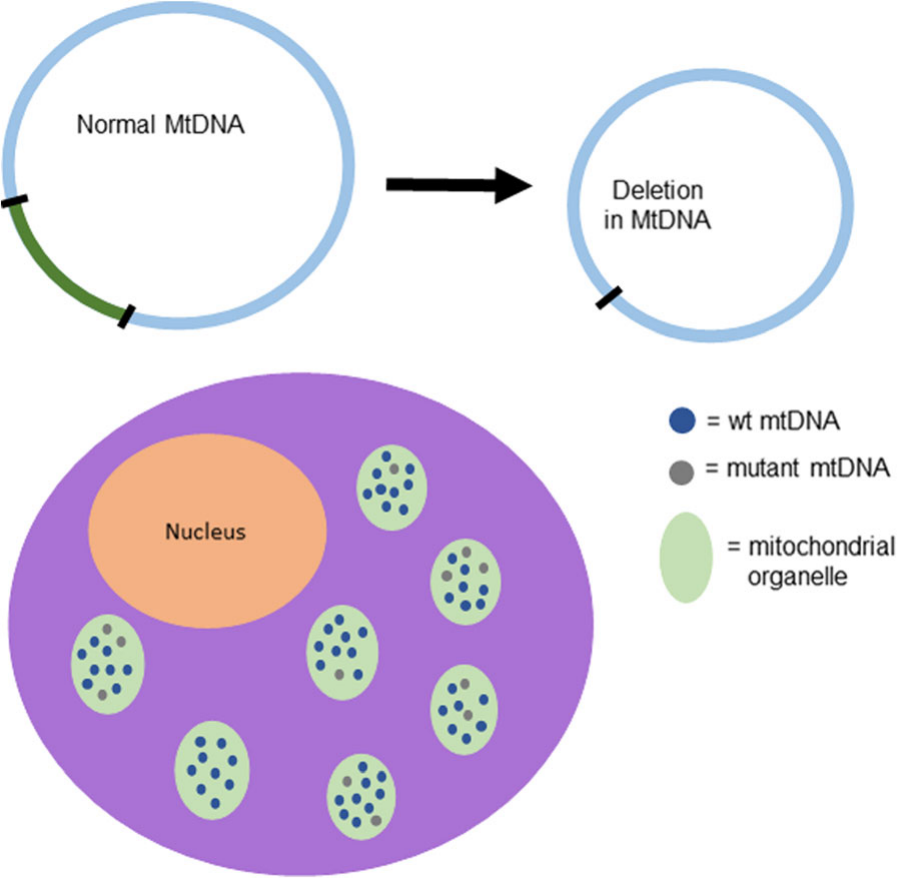
Depiction of a hypothetical mitochondrial genome deletion (top). The intact genome is shown at left with deleted segment indicated in green and a copy harboring the deletion at right. In the cell (bottom), both intact and deletion copies are present within the mitochondrial organelles, with per-cell abundance of the deletion at a low percentage

This study focuses on detecting deletions within the mitochondrial chromosome (Fig. 1). With the advent of genome-wide technologies, a great deal of research has been devoted to developing methodology to identify sub-chromosomal gains and losses from “next-generation” sequencing (NGS) data [10–12]. Few of these approaches have been applied to the mitochondrial genome. One of the reasons for this is the fluidity of mtDNA abundance and content. For instance, although generally only two haplotypes per nuclear chromosome exist in an individual human (the exception being tumor cells), many distinct mitochondrial haplotypes may exist within a single individual, even in the same cell [13]. More than one distinct mtDNA haplotype being present in a single cell, tissue, or individual is known as heteroplasmy. MtDNA chromosomes harboring deletions are often present at very low heteroplasmy levels, making them difficult to detect.

In this paper, we describe MitoDel, the computational procedure we have developed to infer mtDNA deletions and their abundances from NGS data. We assess the theoretical sensitivity of our approach, and test its sensitivity and specificity using simulated data. We apply MitoDel to previously published data from sequencing experiments involving aging human brain tissue, and to the large public 1000 Genomes dataset [14]. We conclude the manuscript with discussion of the results and future directions.

Software implementing MitoDel is available at the LaFramboise laboratory website (http://mendel.gene.cwru.edu/laframboiselab/).

## Methods

### Acquisition of previously-published data

Courtesy of Dr. Sion Williams of the University of Miami, we obtained raw sequence data from the Williams et al. study [15]. Whole-genome .bam files of aligned and unaligned reads were downloaded from the 1000 Genomes website [14].

### Simulated data

Read data from samples harboring deletions of various sizes and heteroplasmy levels were simulated using the ART simulator (version ART-ChocolateCherryCake-03-19-2015). To simulate an experiment generating *R* paired-end Illumina reads from a sample with a given deletion present in proportion *p* of mtDNA copies, we first modified the .fasta file containing the revised Cambridge Reference Sequence (rCRS; NC_012920.1) [16], removing a string of bases corresponding to the desired deletion. We then used ART to simulate (1 – *p*) x *R* reads from the rCRS reference, and *p* x *R* reads from the deleted version.

### Raw read preprocessing

All .fastq files were first aligned to a modified human genome build hg19 using BWA [17]. Hg19 was modified by removing the original chrM and replacing it with the rCRS. Reads were not realigned if a .bam file was available.

### MitoDel’s bioinformatic pipeline to detect mitochondrial DNA deletions

**Fig. 2:**
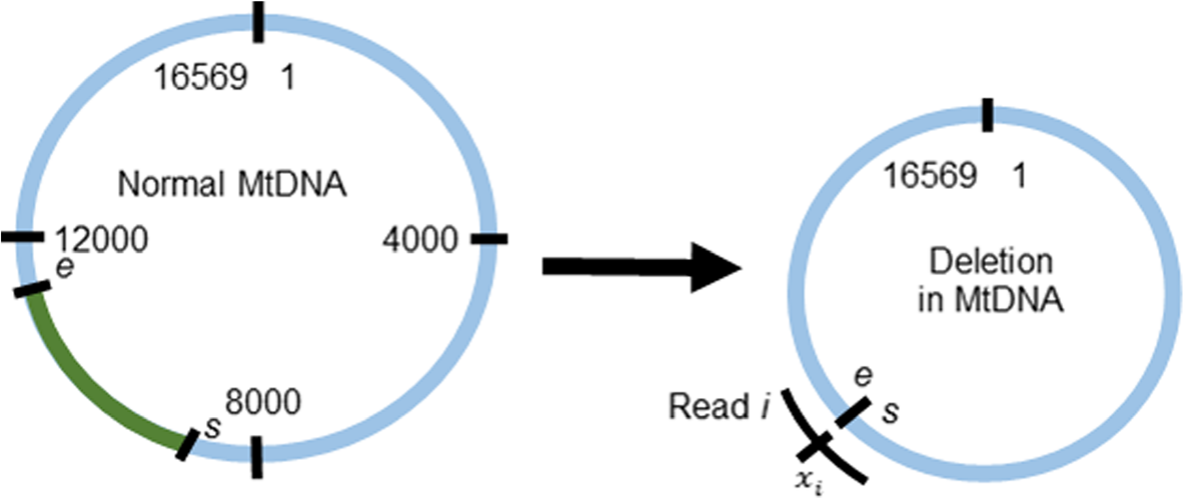
Standard mitochondrial reference genome numbering shown in interior of the circular genome, with the deleted segment, from base position *s* + 1 to base position *e* – 1, indicated in green, and the copy harboring the deletion shown at right. The position *x*_*i*_ in a single hypothetical read *i* (black arc) shown in circle exterior. This read may be unaligned by BWA [17], but BLAT [20] will be able to align its two segments as a split read

The mitochondrial genome is described as circular chromosome 16,569 bases in length. In the reference genome, the base positions are numbered in a clock-like manner, from 5′ to 3′ on the “light” strand, from base position 1 to base position 16,569 (Fig. 2). When a deletion occurs, it has the effect of moving two base positions that are distant in the intact genome to being adjacent. It follows that reads harboring the resulting fusion point will either: i) not be deemed by the standard NGS aligner as having come from the mitochondrial genome, and will therefore be unaligned (Fig. 2); or ii) only be aligned after clipping or other modifications to the read. These modifications will be recorded in the CIGAR string field of the resulting .sam/.bam file [18], and the modified reads may thus be identified. Recovering these sequences and mining them for recurrent fusion points is the procedure that underpins our approach, as briefly described in a published abstract [19]. Furthermore, the relative abundance of mtDNA haplotypes harboring the deletion may be inferred by comparing the number of reads harboring the fusion point with the average read depth across the mitochondrial chromosome.

Formally (notation also shown in Fig. 2), suppose that the region from mitochondrial base position *s* + 1 to base position *e* - 1 is deleted in proportion *p* of mtDNA copies, and suppose that the NGS experiment generates reads of length *l* bases. Suppose further that *n* reads harbor the deletion fusion point. For the *i* ^th^ of these reads, let *x*_*i*_ (*i* = 1,…,*n*) denote the position in the read (oriented from lower mtDNA base position to higher) harboring base position *s* in the mitochondrial genome (1 ≤ *x*_*i*_ ≤ *l*). Many of these reads will not align to anywhere on the reference genome, and will be therefore be marked as “unaligned” in the resulting .bam file output by BWA. We extract these unaligned reads, plus all reads with CIGAR strings indicating potential structural variants. This set of reads is then aligned to rCRS using BLAT [20].

Unlike BWA and other aligners designed for NGS data, BLAT is able to find splits of reads into multiple segments that each align to separate sites in a reference genome. This capability comes at an extremely high computational cost, which is among the reasons that NGS aligners do not include it. However, since the mitochondrial genome is relatively extremely small, and since we filter out reads that map perfectly a priori, we are able to take advantage of BLAT without excessive computational burden (see “Compute time considerations” subsection below).

BLAT’s output for split reads includes the start and end read positions of each aligned segment of the read. In the above notation, this would correspond to two segments with (start, end) positions (1, *x*_*i*_) and (*x*_*i*_ + 1, *l*) for read *i*. BLAT’s output also includes the beginning and ending genomic coordinates (mtDNA base position) to which each segment aligns. In the above notation, this would correspond to mtDNA positions (*s* - *x*_*i*_ + 1, *s*) and (*e*, *e + l - x*_*i*_ − 1) for the two read segments. It follows that we may interrogate the BLAT output for a set of *n* split reads that:

1. each split into two segments,
2. each have both segments map to the same strand of the mitochondrial genome,
3. all suggest the same deleted segment, and
4. collectively have the fusion point appear in at least five different locations in the read, i.e. the set {*x*_1_,…,*x*_*n*_} contains at least five unique elements.

This last requirement helps avoid false positive deletions such as those that are the result of well-known sequencing artifacts such as PCR errors or the aligner splitting a read due to a single nucleotide substitution difference from the reference genome.

If the number of reads suggesting precisely the same breakpoint is sufficiently large, enough evidence is deemed to have been produced to report the breakpoint as biologically real. This number *n* (where a deletion is called if at least *n* split reads support it) is a tuning parameter. Clearly, higher values of *n* will increase specificity and decrease sensitivity. We use *n* = 10 as a default value in MitoDel (see “Results from simulated reads” subsection below for justification), though this may be adjusted in the corresponding software.

**Fig. 3:**
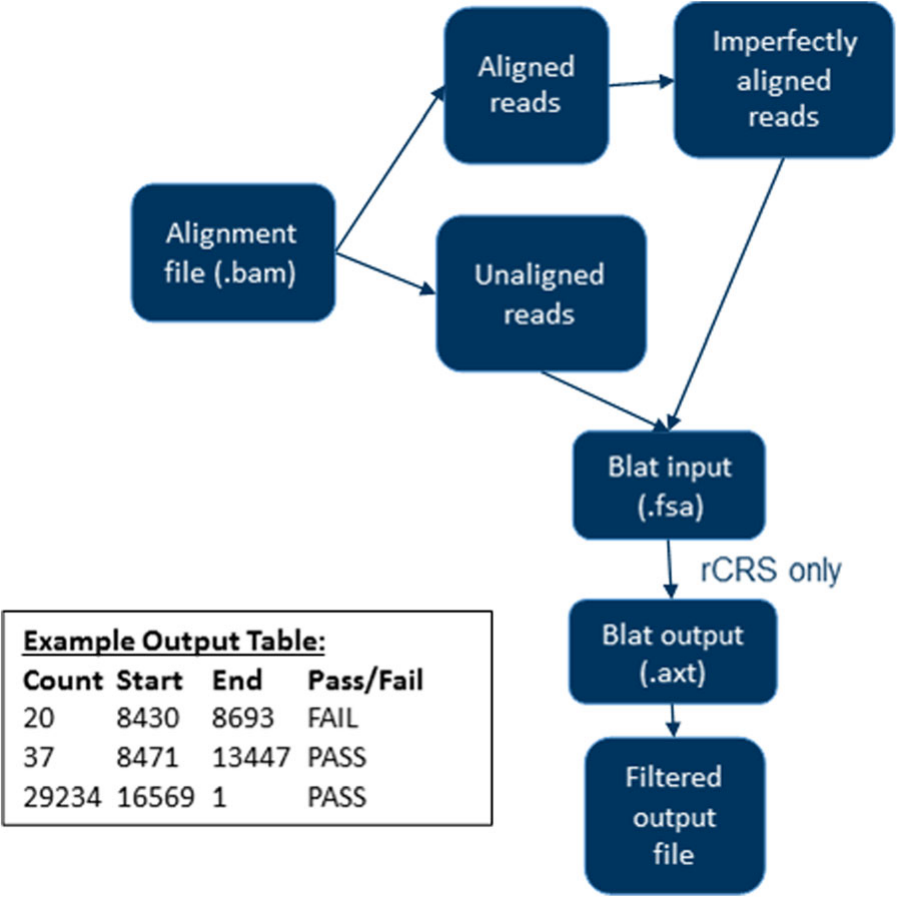
An overview of MitoDel, from aligned sequence files to mtDNA deletion fusion point and abundance inferences. A sample output table from the software is shown at bottom, where each row is a putative deletion with read support, deleted segment coordinates, and indication of whether it passes quality filtering

An overview of the MitoDel procedure is shown in Fig. 3.

### Estimating heteroplasmy level and confidence interval

The number *N* of reads harboring a deletion given the total reads in the experiment would be expected to approximately follow a binomial distribution Bin(*r*, *q*), where *r* is the number of reads from the mitochondrial genome, and *q i*s the proportion of reads that harbor the deletion. Since there are 16,569 possible starting positions for mtDNA reads, reads from the deleted copy of the genome will harbor the deletion with probability *l*/16569, where *l* is the length of the reads. Therefore, if we estimate *q* as $\widehat{q}=n/r$ we may estimate the heteroplasmy level as $\widehat{q}\times \frac{16569}{l}$. The 1 – α confidence intervals on *q* may be computed analytically [21] as

$$ \left(\frac{1}{1+\frac{r-n+1}{n}{F}_{2\left(r-n+1\right),2n,\frac{\alpha }{2}}},\frac{\frac{n+1}{r-n}{F}_{2\left(n+1\right),2\left(r-n\right),\frac{\alpha }{2}}}{1+\frac{n+1}{r-n}{F}_{2\left(n+1\right),2\left(r-n\right),\frac{\alpha }{2}}}\right), $$

where *F*_*a,b,c*_ denotes the 1 – c quantile of the *F* distribution with *a* and *b* degrees of freedom. We can then transform this confidence interval on *q* to a confidence interval on the heteroplasmy level.

## Results

### Theoretical sensitivity

As mentioned above, mtDNA deletions have typically been observed at extremely low abundances, frequently a fraction of 1 %. Therefore, sequencing at high read depths is necessary to detect deletions. When designing NGS experiments for this purpose, researchers also must take into account the high numbers of nuclear genome reads present in the sequencing data, which will decrease the average number of reads per mitochondrial base position. Indeed, unless an mtDNA enrichment procedure is applied in the laboratory prior to DNA sequencing, only approximately 0.2% of DNA is expected to be mitochondrial [22]. Even with enrichment, the sensitivity of our computational procedure clearly depends on the number of mtDNA reads, which is a function of the enrichment protocol’s efficiency. We and others have performed studies developing and comparing various mtDNA enrichment protocols [22, 23], with varying results depending on the tissue type and other factors. Theoretical sensitivity for a sequencing experiment therefore here takes into account various enrichment levels.

Given an NGS experiment with *M* total reads and mtDNA enrichment level *E* (enrichment here is defined as the proportion of DNA in the sample that is mitochondrial as opposed to nuclear), the number of reads harboring a given mitochondrial base position is expected to be approximately

$$ N\approx \left(M\times E\times l\right)/16569. $$

Computations using the binomial distribution show, for example, that a typical run on a standard Illumina MiSeq of ~50 million reads from a sample subjected to a protocol yielding 60% mtDNA enrichment would allow for detection of deletions as low as 0.006% with 95% probability, using our default threshold of 10 reads supporting the deletion. Sequencing experiments with higher numbers of reads and/or better enrichment protocols could find even lower-level deletions.

### Results from simulated reads

We simulated sequencing experiments for three different mitochondrial deletions (small, medium, and large), generating paired-end Illumina reads of 150 bp each, with mean 300 and standard deviation 100 for the distance between reads. The simulated deletions were 15 bp (m.700_715del), 200 bp (m.5000_5200del), and one comparable in size to the well-known “common deletion” [24] at 4900 bp (m.6930_11830del). For each deletion, 100 replicates of the corresponding simulated sequencing experiment were run. Each iteration sampled the deleted genome at 70× coverage and the intact mitochondrial genome at 69930× coverage, thereby simulating a heteroplasmy level of 0.1%, yielding paired-end .fastq files. These files were aligned and then run through MitoDel. We also performed 100 iterations of a simulation sampling the genome with the 200 bp deletion at 700× coverage and the intact mitochondrial genome at 69300× coverage, thereby simulating a heteroplasmy level of 1%.

**Fig. 4:**
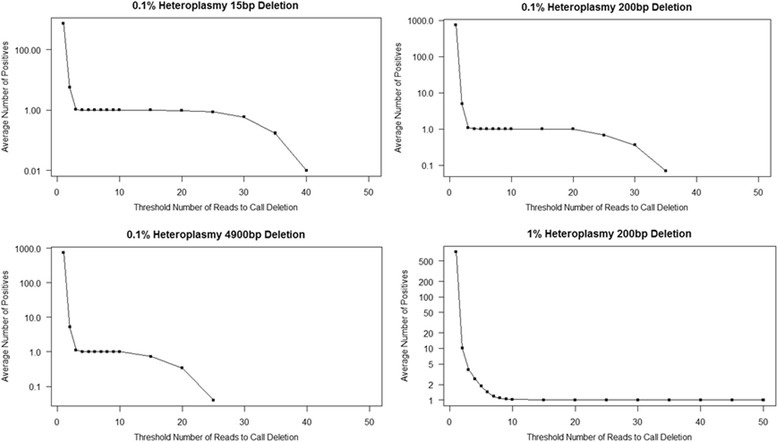
False positive rates and sensitivity of MitoDel. Vertical axis (log scale) indicates the average number of deletions called with a least *n* reads supporting the deletion, where *n* is indicated on horizontal axis. Each experiment simulates one actual deletion, so average positives greater than 1.0 are false positives, while average positives less than one indicate specificity. Average positives exactly 1.0 indicate perfect sensitivity and specificity

We used the simulations to assess the sensitivity and the false positive rate of MitoDel, varying the number of reads harboring a deletion necessary for it to be called (the *n* parameter from above) from 1 to 50. For each value of *n*, we computed the proportion of the 100 replicates calling the deletion at at least that threshold. This value was used as an estimate of MitoDel’s sensitivity. We also tallied the average number of deletions called (all but one of which are false positives since we only “spiked in” one deletion at a time) across the iterations for each threshold *n*. This was used to estimate the average number of false positives. These averages, (Fig. 4) show that for a variety of deletions at very low heteroplasmy levels, MitoDel remains highly specific. The average number of false positive calls falls steeply with increasing threshold *n* until about 10 reads, a threshold at which all called deletions are true positives, and the true deletion is always called. These simulation experiments led us to choose 10 as the default value for *n* in the MitoDel software.

### Detection of low-level deletions in brain tissue

We applied MitoDel to NGS data generated from human brain tissue for a previously-published study [15]. Using a method with no software and few computational details provided, the authors analyzed tissue from young (< 35 years old) and aged (> 66 years old) individuals. The study reported a ~ 5000 bp deletion (m.8483_13459del4977, the well-known common deletion [24]) present in the majority of the aged individuals but a minority of young individuals.

We acquired the raw sequencing data for 10 of these individuals directly from the authors and applied MitoDel agnostically, without targeting the common deletion specifically. Our presence/absence largely agreed with the authors’ assessments, except that we found evidence for a low-abundance deletion (0.58%) in an individual that the Williams et al. study deemed absent of deletions (Table 1). Manual inspection of the reads gives evidence that the deletion is indeed present, and our simulation results suggest that a false positive is unlikely. Generally, the heteroplasmy levels reported in the Williams et al. study were lower than, but were correlated with, our inferences.

**Table 1:** Application of MitoDel to data from [15]

Individual ID	Williams et al. Reported Heteroplasmy Level	MitoDel Heteroplasmy Level (95% CI)
55–10 (Y11)	0%	0%
56–10 (Y12)	0.15%	1.79% (1.52%,2.10%)
57–10 (Y13)	0.015%	0.28% (0.17%, 0.44%)
58–01 (Y15)	0%	0%
59–01 (A16)	0.4%	5.15% (4.58%, 5.77%)
60–10 (A17)	0.1%	1.35% (1.11%, 1.62%)
61–10 (A18)	0.1%	1.11% (0.88%, 1.38%)
62–10 (A19)	0.15%	2.79% (2.42%, 3.21%)
77–10 (Y3)	0%	0.58% (0.33%, 0.98%)
78–10 (Y4)	0%	0%

Individual IDs beginning with Y indicate young individuals, and those beginning with A indicate aged individuals

### MtDNA deletions in 1000 genomes data

**Fig. 5:**
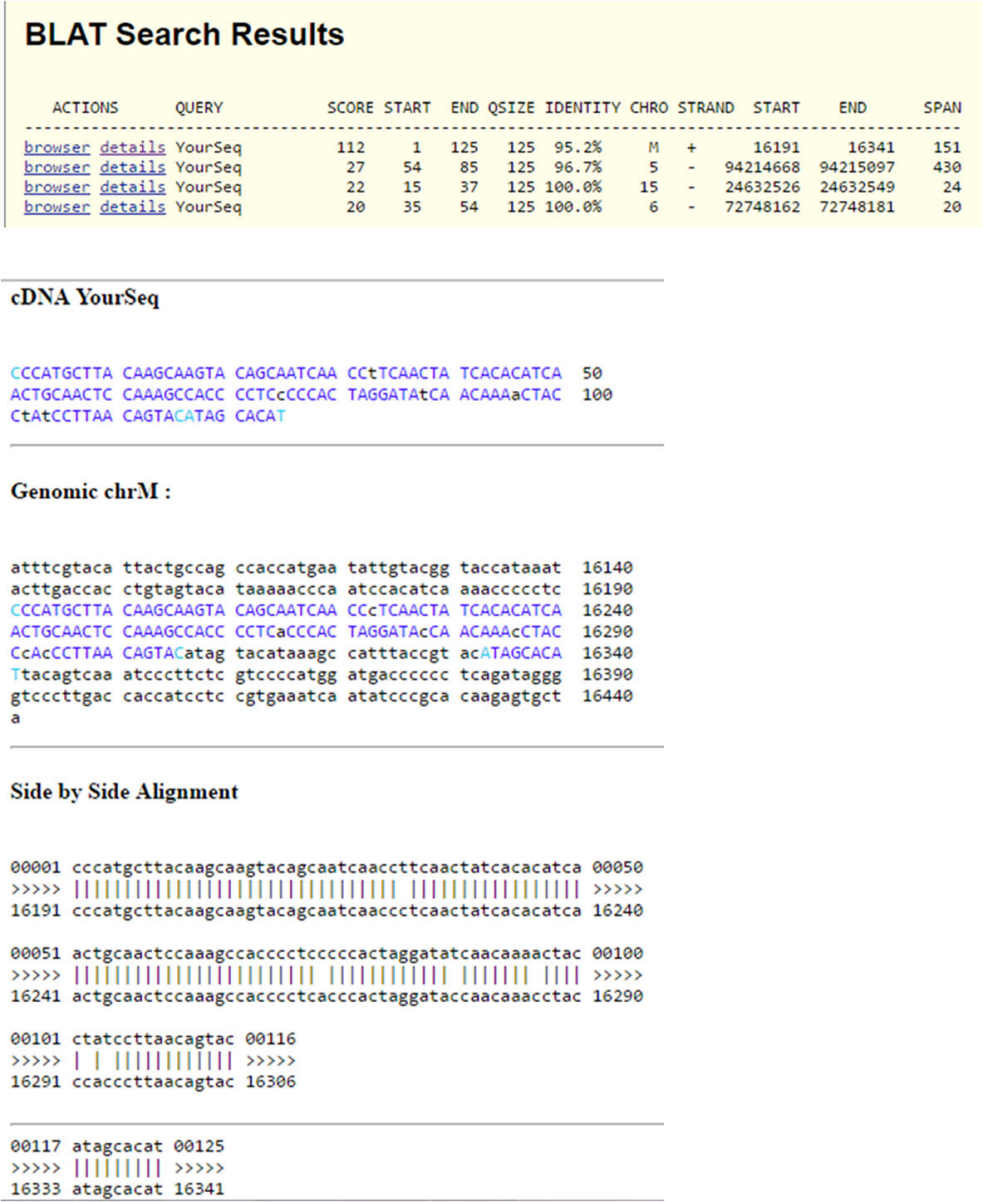
BLAT output showing the split alignment of a read harboring a putative 27 bp deletion in 1000 Genomes individual HG02332

Applying MitoDel to 10 .bam files from phase 3 of the 1000 Genomes Project, we found a 27-bp deletion (m.16306_16333del27) in the D-loop of individual HG02332. A total of 96 reads were found supporting this deletion, with 1,115,366 reads aligning to the chrM. The estimated heteroplasmy level of the deletion is therefore 0.71% with 95% confidence interval (0.58%, 0.86%) as calculated above. This deletion has not previously been reported, according to the mitochondrial deletion database MitoBreak [25]. However, as Fig. 5 shows with a representative read, the reads are consistent with the deletion and do not match well to any autosomal region, and therefore the deletion call is unlikely to be a false positive.

### Compute time considerations

On a Dell PowerEdge R630 with two 2.3GHz Intel Xeon E5–2670 v3 processors and 256 GB of RAM, a .fastq file with 16 million 100 bp paired end reads took approximately 141 min to run. Therefore, MitoDel can easily handle raw sequence files of the sizes that will be routinely generated for the foreseeable future.

## Discussion

MitoDel is methodologically very straightforward. It relies on the highly accurate split-read mapping capabilities of BLAT, which would be far too computationally expensive to use in whole-genome applications. We are able to take advantage of these capabilities by first omitting all reads that mapped well to the human genome, thereby greatly reducing mapping burden for BLAT. The fact that the mtDNA reference genome is so small compared to the nuclear genome also reduces the burden. As demonstrated by our simulation results, the high precision of BLAT split read mapping confers extremely favorable precision and recall levels to MitoDel, even without more sophisticated statistical modeling of sequencing errors, mapping errors, and other sources of noise.

To our knowledge, there have been three methods published that could conceivably be used to detect low-level mtDNA deletions specifically. Mitoseek [26] is designed to detect all types of mitochondrial DNA-level variation. However, its deletion tool only reports read pairs whose mapped distance apart exceeds a user-specified threshold. It does not actually call the deletions or specify their coordinates, and therefore cannot be directly compared with MitoDel for accuracy. Delly [10] is designed to detect structural variants in a cancer context. While it allows for polyploidy when making calls, it does not handle heteroplasmy levels below 1% as MitoDel does. We were unable to successfully install and run the third method, MToolBox [27].

## Conclusions

Here we have presented a computational method, MitoDel, to detect and quantify mtDNA deletions from next-generation sequencing experiments. Our method meets a need for software to identify aberrations present at extremely low levels. Our results demonstrate the ability to call deletions present at well below 1% heteroplasmy levels, with a very low false positive rate. Similar methods for detection of chromosomal aberrations have been developed for the nuclear genome, but these are tuned for much higher abundances. Indeed, a deletion in the nuclear genome will be present at at least 50% abundance in a cell. In heterogeneous tumor samples, the level may be lower in the overall sample, but available methods are not suitable for the extremely low abundances (< 1%) that MitoDel targets.

We can see a number of extensions to the work presented here. The most obvious would be to detect other types of mitochondrial chromosomal aberrations such as tandem duplications and inversions. Although these classes of aberration have rarely been described in mtDNA, only newer technology can detect them when present at low abundances, which could explain the lack of prior studies reporting them. Even mitochondrial-nuclear translocations have recently been described in cancer samples [28]. Theoretically, the approach described here could be easily modified to detect all of these lesion types.

## Abbreviations

mtDNA: Mitochondrial DNA
NGS: Next-generation sequencing
rCRS: Revised Cambridge reference sequence
